# Delayed or failure to follow-up abnormal breast cancer screening mammograms in primary care: a systematic review

**DOI:** 10.1186/s12885-021-08100-3

**Published:** 2021-04-07

**Authors:** Jeanette C. Reece, Eleanor F. G. Neal, Peter Nguyen, Jennifer G. McIntosh, Jon D. Emery

**Affiliations:** 1 Colorectal Cancer Unit, Centre for Epidemiology and Biostatistics and Neuroepidemiology Unit, Centre for Epidemiology and Biostatistics, Melbourne School of Population and Global Health, The University of Melbourne, Level 3 207 Bouverie Street, Parkville, VIC 3010 Australia; 2 Centre for Cancer Research, Victorian Comprehensive Cancer Centre, Faculty of Medicine, Dentistry and Health Sciences, The University of Melbourne, Melbourne, Australia; 3 Infection and Immunity, Murdoch Children’s Research Institute, Parkville, Australia; 4 Centre for International Child Health, Department of Paediatrics, The University of Melbourne, Parkville, Australia; 5 Department of General Practice, Faculty of Medicine, Dentistry and Health Sciences, The University of Melbourne, Melbourne, Australia; 6 Department of Software Systems and Cybersecurity, Faculty of Information Technology, Monash University, VIC Clayton, Australia

**Keywords:** Primary care, Breast cancer screening, Abnormal mammogram, Inadequate follow-up

## Abstract

**Background:**

Successful breast cancer screening relies on timely follow-up of abnormal mammograms. Delayed or failure to follow-up abnormal mammograms undermines the potential benefits of screening and is associated with poorer outcomes. However, a comprehensive review of inadequate follow-up of abnormal mammograms in primary care has not previously been reported in the literature. This review could identify modifiable factors that influence follow-up, which if addressed, may lead to improved follow-up and patient outcomes.

**Methods:**

A systematic literature review to determine the extent of inadequate follow-up of abnormal screening mammograms in primary care and identify factors impacting on follow-up was conducted. Relevant studies published between 1 January, 1990 and 29 October, 2020 were identified by searching MEDLINE®, Embase, CINAHL® and Cochrane Library, including reference and citation checking. Joanna Briggs Institute Critical Appraisal Checklists were used to assess the risk of bias of included studies according to study design.

**Results:**

Eighteen publications reporting on 17 studies met inclusion criteria; 16 quantitative and two qualitative studies. All studies were conducted in the United States, except one study from the Netherlands. Failure to follow-up abnormal screening mammograms within 3 and at 6 months ranged from 7.2–33% and 27.3–71.6%, respectively. Women of ethnic minority and lower education attainment were more likely to have inadequate follow-up. Factors influencing follow-up included physician-patient miscommunication, information overload created by automated alerts, the absence of adequate retrieval systems to access patient’s results and a lack of coordination of patient records. Logistical barriers to follow-up included inconvenient clinic hours and inconsistent primary care providers. Patient navigation and case management with increased patient education and counselling by physicians was demonstrated to improve follow-up.

**Conclusions:**

Follow-up of abnormal mammograms in primary care is suboptimal. However, interventions addressing amendable factors that negatively impact on follow-up have the potential to improve follow-up, especially for populations of women at risk of inadequate follow-up.

**Supplementary Information:**

The online version contains supplementary material available at 10.1186/s12885-021-08100-3.

## Background

Breast cancer is the most commonly diagnosed cancer and a leading cause of cancer-related death among women worldwide [1]. The standard of care for breast cancer screening is digital mammography, which is associated with a 20% reduction in breast cancer-related mortality in women at average risk of breast cancer [2, 3]. Mammographic screening relies on the follow-up of abnormal (potentially clinically significant) mammograms in a timely manner. Delays in follow-up may compromise the prognostic benefits of screening, [4, 5] and lead to increased emotional distress and anxiety [6].

Breast screening guidelines in the United States (US) and Europe recommend women receive notification of abnormal mammogram results within five days of the primary care provider’s (PCP’s) receipt of results [7, 8]. In Australia and the Netherlands, clinical guidelines recommend women should receive mammogram results within 28 days and 14 days of screening, respectively [9, 10]. To guide follow-up, American College of Radiology (ACR) Breast Imaging Reporting and Data System® (BIRADS®) is used to classify mammograms, with highly suggestive of malignancy (BIRADS®-5), suspicious malignancy (BIRADS®-4) or indeterminant (BIRADS®-0) mammograms recommended immediate (within 3 months) follow-up and likely benign (BIRADS®-3) mammograms recommended short term (3–6 months) follow-up [7, 11].

In many healthcare systems, especially in the US, primary care providers (PCPs) play a critical role in promoting and encouraging patient participation in preventative health services, including mammography screening, as well as organising these preventative services [12]. In particular, PCPs have an important influence on providing different modalities of breast cancer screening across women of all ages, either as organised (population-based) mammography screening usually for women over 50 years or non-organised patient or PCP-driven (opportunistic) screening [13]. Moreover, PCPs act as “gate-keepers” to secondary care for the diagnostic assessment of abnormal mammograms to ensure timely follow-up to diagnostic resolution, [14] but are also responsible for informing patients of abnormal mammogram results, potential impacts on patients’ health status and recommended follow-up investigation and critically, timely intervention [11, 15]. Despite this, several studies report that follow-up of abnormal mammogram results in primary care is suboptimal, with delayed follow-up associated with poorer patient morbidity and mortality outcomes [4, 5]. The extent of inadequate follow-up in primary care and factors influencing follow-up has not been well-studied, however, there is evidence to suggest delayed follow-up is due to health system-, PCP- and patient-related barriers [16–27].

Despite the vital role PCPs play in breast screening (both organised and non-organised), particularly in the US, the effectiveness of follow-up after abnormal mammography in primary care has not been well-studied. This present study aimed to systematically review the evidence related to inadequate follow-up of abnormal screening mammograms among women in primary care and identify factors influencing the follow-up of abnormal screening mammograms in primary care. Increased understanding of the extent of inadequate follow-up and barriers to follow-up will enable primary care-specific targeted interventions addressing barriers to inadequate follow-up to be devised, which in turn may improve follow-up and ultimately, patient outcomes, particularly for women identified as being at the highest risk of inadequate follow-up.

## Methods

A systematic review of relevant studies was conducted using the Preferred Reporting Items for Systematic Reviews and Meta-Analyses (PRISMA) criteria [28]. This review was registered in PROSPERO (Registration ID: CRD42019139517).

The ACR BIRADS® reporting tool was used to define abnormal or clinically significant mammograms (BIRADS®-0, BIRADS®-3, BIRADS®-4 or BIRADS®-5) [7].

### Search strategy

Medical Subject Headings (MeSH) terms were used to search four databases: EMBASE, MEDLINE via Ovid, the Cochrane Central Register of Controlled Trials, and Cumulative Index of Nursing and Allied Health Literature (CINAHL). The search strategy was an intersection of MeSH terms referring to “family practice” or “primary care”, “delay”/“follow-up”/“errors” and “screening”/“cancer screening” tests for breast, colorectal, gynaecological, prostate, lung, liver and skin cancer to capture all relevant articles related to inadequate follow-up of abnormal tests results for these cancers to enable a series of systematic reviews to be performed examining inadequate follow-up for each respective cancer. Studies pertaining to inadequate follow-up (failure to follow-up, delayed follow-up or inappropriate follow-up) of abnormal screening mammograms in primary/community/ambulatory/family practice settings were specifically selected for this review via relevant abstracts identified and independently reviewed by two co-authors (P.N., J.C.R.). The full electronic search strategy is available in Supplementary Table 1.

Full text articles that fulfilled the study criteria were identified by two independent reviewers (J.C.R., E.F.G.N.), and included manual reference and citation checking to identify relevant studies not found from the search. Studies were included if they specifically examined inadequate follow-up after abnormal screening mammogram. Studies that exclusively examined appropriate/timely follow-up as the outcome were excluded. This decision was made to avoid making the potentially incorrect assumption that women that did not have adequate follow-up equated to inadequate follow-up as these women may have accessed care elsewhere.

### Data abstraction

Data from full text articles were independently abstracted and evaluated by two co-authors (J.C.R. and E.F.G.N.). Any discrepancies between reviewers were discussed and resolved by consultation with a third reviewer (J.D.E.). A standardized data extraction form was used to confirm study eligibility, evaluate study and participant characteristics and extract data from included studies [29].

### Inclusion and exclusion criteria

Inclusion criteria consisted of studies:

- published between 1 January, 1990 and 29 October, 2020.
- conducted in primary care or a US community-based setting that included family practice, internal medicine or obstetrics/gynaecology services in public or private facilities provided ≥80% was in primary care
- examined inadequate abnormal mammogram follow-up of breast cancer screening mammograms (not diagnostic mammograms)

Studies were excluded if they:

- exclusively examined timely follow-up of abnormal mammograms but did not measure inadequate follow-up
- included women with a current or prior history of breast cancer
- examined follow-up of clinical symptoms and mammograms collectively
- did not delineate abnormal mammogram follow-up in the context of examining multiple cancers collectively
- examined follow-up of diagnostic mammograms
- were not in English
- were unpublished work, academic theses or conference abstracts
- were case studies, reviews, protocols or editorials
- were studies involving men with breast cancer
- were studies that exclusively examined inadequate follow-up resulting in malpractice claims due to the high selection bias of study participants

### Assessment of risk of bias

Studies were assessed for risk of bias by two reviewers (E.F.G.N. and J.C.R.) using the Joanna Briggs Institute (JBI) Critical Appraisal Checklists, [30] using appropriate checklist for study type:

- cross-sectional
- cohort
- randomised control trial (RCT)
- qualitative research

The JBI tool comprises 8–12 questions, with possible answers “yes”, “no”, “unclear” or “not applicable” depending on study type. To define the quality of studies, questions assessed as low risk of bias were divided by the total number of questions to determine a percentage score. Studies were classified as low (> 80%), moderate (60–80%) and high risk (< 60%) of bias prior to commencing risk appraisals [31, 32]. Studies were not excluded based on their risk of bias to ensure transparency and completeness of reporting findings from all studies identified as relevant for the review as recommended by Shea et al. [33].

The percentage of women with abnormal mammograms that had inadequate follow-up in each study was extracted. We reported factors positively or negatively associated with follow-up (inadequate or adequate) to provide a comprehensive picture of all barriers and facilitators of follow-up. Principal summary measures are described as reported in each eligible study.

A meta-analysis was not performed due to the heterogeneity of the data in the included studies, instead a narrative review of results in the eligible studies was conducted.

## Results

**Fig. 1:**
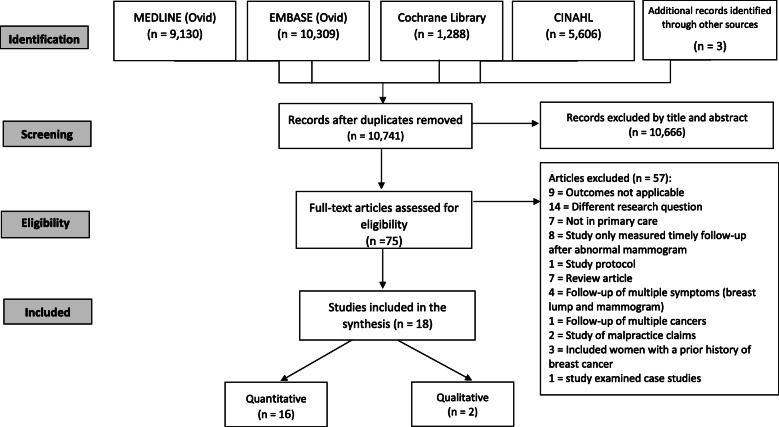
PRISMA flow diagram of inclusion of studies

The search strategy identified 10,741 titles, of which 75 full text articles were reviewed for eligibility (Fig. 1). Eighteen articles reporting on 17 individual studies were included in the systematic review.

Characteristics of included studies chronologically ordered by publication date are outlined in Table 1. Studies comprised one randomised controlled trial (RCT), [34] 11 cohort, [16, 18, 19, 21, 22, 24, 25, 35–38] two cross-sectional, [23, 27], two qualitative studies, [39, 40] and two mixed method studies (cross-sectional and qualitative, [17] and cohort and qualitative) [26]. All studies were US-based, except one cohort study from the Netherlands [26].

**Table 1:**
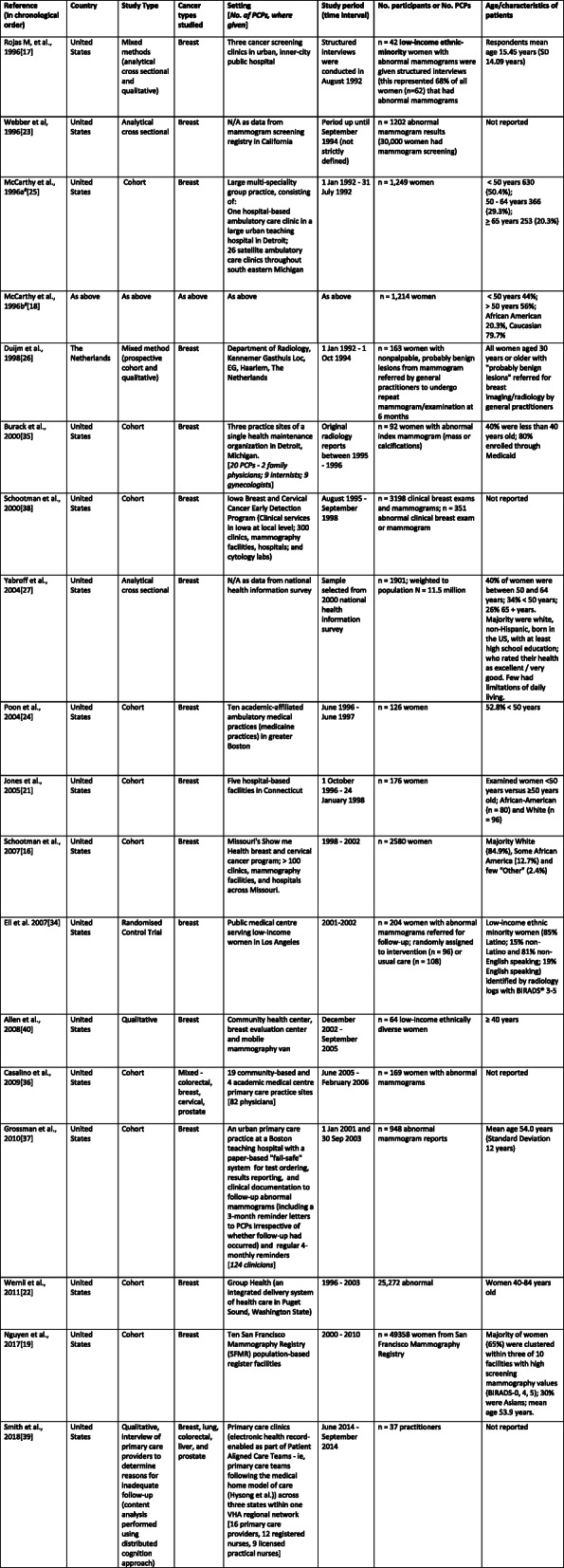
Characteristics of included studies

JBI risk of bias assessments identified 10 articles at low risk of bias, seven at moderate risk and one at high risk (Table 2, Supplementary Table 2).

**Table 2:**
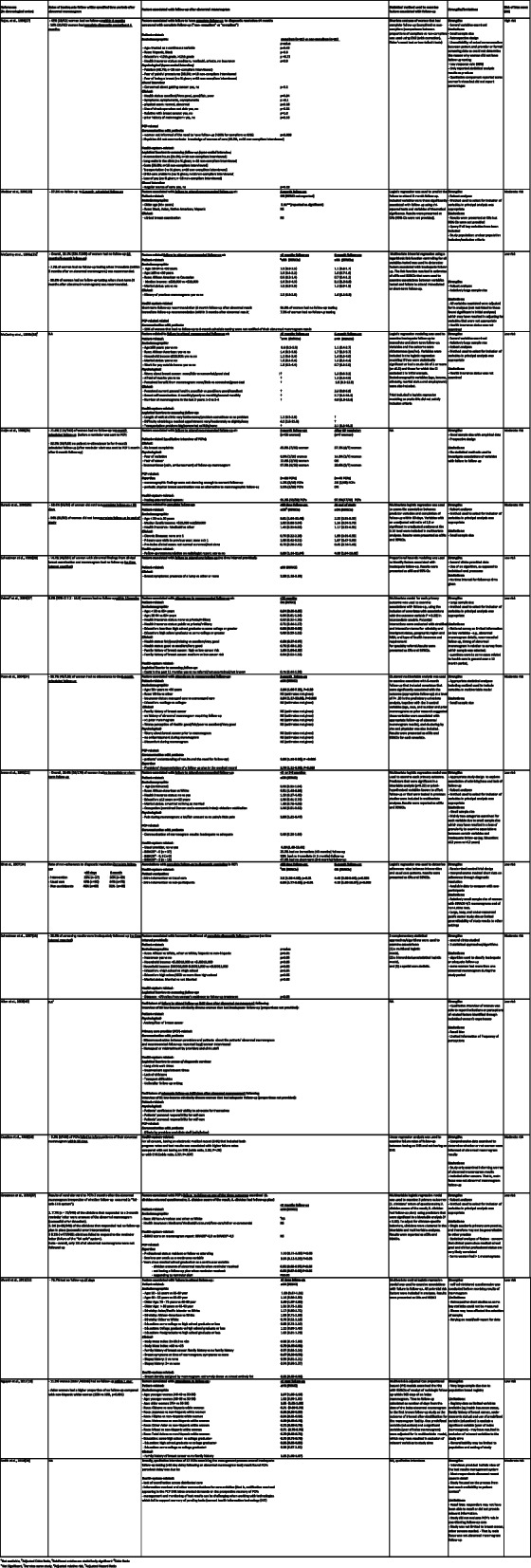
Summary of findings from included studies

Study findings are summarised in Table 2. The definition of inadequate follow-up following abnormal mammogram varied across studies and included:

- Failure to attend scheduled/recommended follow-up, [18, 21, 23, 25, 26, 41] or any follow-up within a specified time, [19, 22, 27, 38–40]
- Failure to undergo complete follow-up to diagnostic resolution, [16, 17, 34, 35]
- Failure of PCP to inform patient of abnormal mammogram result, [36] and
- PCP failed to acknowledge follow-up letter, was not aware of result or had no follow-up plan [37].

Measures of follow-up after abnormal mammogram included specialist referrals and/or attendance, diagnostic imaging and/or fine needle biopsy, open surgical biopsy or undergo complete follow-up to diagnostic resolution as per recommended guidelines [42, 43].

### Rates of inadequate follow-up

Rates of inadequate abnormal mammogram follow-up are presented in Table 2. Studies are ordered chronologically by publication date to reflect any reduction in inadequate abnormal mammogram follow-up that has occurred over time, particularly delays in the complete follow-up to diagnostic resolution, due to the implementation of advanced technology that has increased diagnostic accuracy and reduced false positives [44]. These innovations include the replacement of screen-film mammography with full-field digital mammography after 2009 and the introduction of new diagnostic modalities including 3D ultrasonography, advanced MRI techniques, and core biopsies to replace fine needle aspiration.

Ten studies examined *failure to attend any follow-up* after an abnormal breast screen result [19, 21, 23–27, 37]. Two of these studies reported rates of 7.2–33% non-attendance within a 3 month follow-up period, [21, 25] and four studies reported rates of 27.3–71.6% non-attendance to 6-monthfollow-up [23–26]. Yabroff et al. and Nguyen et al. found 8.6%, [27] and 11.3%, [19] of women, respectively, had not attended follow-up within one year. One study described a “fail-safe” system where only 1% of women with abnormal mammogram results failed to attend follow-up [37].

Two studies examined *failure to undergo complete diagnostic follow-up.* The first, a retrospective analysis of clinical records, found 68.4% of women had incomplete follow-up within 60 days of an abnormal mammogram and 34% after 11 months [35]. The second was an RCT: The SAFe (Screening Adherence Follow-up) trial, where the SAFe intervention comprised patient navigation/case management intervention with increased education and counselling that aimed to reduce rates of incomplete diagnostic follow-up. Inadequate follow-up between control and intervention groups was 43 and 23% (*p* = 0.01), respectively, after 60 days, and 34 and 10% (*p* < 0.001), respectively, after 8 months [34].

Two qualitative studies did not report rates of inadequate follow-up but explored barriers and facilitators to abnormal mammogram follow-up [39, 40].

### Factors contributing to abnormal mammogram follow-up

Factors influencing follow-up of abnormal mammograms were classified into health system-, PCP- or patient-related factors (Table 2).

### Health system-related factors

#### *Electronic health record (EHR)*

Two studies examined the use of EHRs to manage the follow-up of abnormal mammograms with comparable results [36, 39]. Qualitative interviewing of 37 PCPs found current health information technology that supported the notification of abnormal cancer screening results, including mammograms, created information overload in PCPs’ EHR inbox and contributed to inadequate follow-up [39]. Similarly, Casalino et al. found EHRs that included both test results and patient progress notes were associated with inadequate follow-up compared with having no EHR for a number of abnormal test results examined, including abnormal mammograms [Odds Ratio (OR) = 2.37; *P* = 0.007) [36].

#### Coordindation between healthcare systems

Smith et al. found PCPs perceived that a lack of coordination across distributed healthcare services, including insufficient information management across organizations and ambiguity about their responsibility in follow-up, contributed to inadequate follow-up [39].

#### *Reminders*

Two studies provided evidence to support the effectiveness of reminders to improve follow-up [26, 37]. In the first study, abnormal mammograms were flagged and internally tracked by the radiology department who were responsible for notifying patients of abnormal results by letter and arranging follow-up tests [37]. In the event the radiology department could not contact the patient, the referring clinician was contacted for assistance. This proved to be a highly effective method for following up women, with only 1% of women not followed up, and at 3 months only 7.7% of PCPs were unaware of the abnormal mammogram. In the second study, radiologists sent a reminder to PCPs if women were overdue for their six monthly follow up and resulted in a reduction of non-attendance to follow-up from 71.6 to 32.5% [26].

#### *Retrieval of patient data*

The study by Duijm et al. sent a questionnaire with reminders to PCPs to explore why the patients had not attended follow-up [26]. Failure to follow-up patients was reportedly clinician driven rather than patient driven, with greater than 90% of PCPs perceiving the absence of adequate retrieval systems to access patient’s results was the main reason for inadequate follow-up (both before and after PCPs received a 6-monthfollow-up reminder).

#### *Patient navigation/case management*

In the SAFe RCT, women in the intervention group received an individualised nurse-delivered patient navigation/case management intervention that included telephone-based health education and counselling based on their risk, reminders and referral to community resources. The results supported the potential of patient navigation to improve abnormal mammogram follow-up; women with BIRAD®-4/− 5 mammograms enrolled in SAFe were 2.5 times more likely to have complete follow-up within 60 days than women in usual care (95% CI 1.36–4.59), with women with BIRAD®-3 mammograms enrolled in SAFe 4.5 times more likely to complete follow-up within 8 months compared with women in usual care (95% CI 2.08–9.64; *P* < 0.001) [34].

#### *Logistical barriers to access follow-up*

Qualitative interview of women who had inadequate follow-up identified inconvenient appointment hours, [17, 40] lengthy clinic waiting times, [17, 40] loss of income, [17] costs, [17] transportation issues, [17, 40] childcare problems, [17, 40] and follow-up in unfamiliar settings as logistical barriers to follow-up [40]. Duijm et al. found around one-quarter of women failed to have follow-up because having another mammogram was inconvenient [26].

In contrast, quantitative studies found no association between clinic waiting times [problematic vs non-problematic; (Adjusted Risk Ratio) ARR = 1.1; 95%CI 0.5–2.8)], [18] transportation problems (big/some vs little/non; ARR = 3.1; 95%CI 0.5–18.3), [18] or costs (referral vs no referral; OR = 0.75; 95%CI 0.44–1.29), [27] and follow-up but living less than 70 miles to follow-up care increased the likelihood of follow-up (*P* < 0.05) [16]. Difficulty obtaining a medical appointment increased the likelihood of non-attendance within 3 months by 4.1-fold (95%CI 1.5–11.3) [18]..

Two smaller studies examined the association between usual care and follow-up with conflicting findings: Jones et al. found an association between failure to attend follow-up and not having a usual PCP [Adjusted odds ratio (aOR) = 4.29; 95%CI 1.48–12.42)], [21] whereas in the quantitative component of the Rojas et al. study, regular versus irregular care was not associated with follow-up (*P* = 0.28) [17].

#### *Radiology report comments*

Two of three studies found comments on radiology reports influenced follow-up (Table 2) [21, 35]. Burack et al. found inclusion of a specific follow-up recommendation by the radiologist in the index mammogram report compared with no recommendation was associated with increased follow-up within 60 days (aOR = 3.55; 95%CI 1.14–11.04) and at least 11 months after the index mammogram (aOR = 4.58; 95%CI 1.54–13.62) [35]. Similarly, non-attendance was higher when short-termfollow-up was recommended compared with immediate follow-up. MCarthy et al. (1996a) found 7.2% of women had no follow-up testing when immediate follow-up was recommended whereas 36.8% of women had no follow-up testing when short-termfollow-up was recommended [25]. Jones et al. found non-attendance to 3–6 months follow-up for BIRADS®-3 mammograms was 47.6%, whereas non-attendance within a 3-monthfollow-up period for BIRADS®-0 and BIRADS®-4/− 5 was 25.5 and 33%, respectively [21]. Conversely, Grossman et al. found no difference in non-attendance to follow-up for BIRADS®-0/− 3 vs BIRADS®-4/− 5, [37] but this is not surprising given immediate follow-up is recommended for BIRADS®-0 and BIRADS®-4/5 [7, 11, 21]. Wernli et al. found extremely dense compared with ‘almost entirely fat’ mammograms were less likely to have delayed follow-up within 7 days compared with at least 7 days (OR = 0.82; 95%CI 0.69–0.96) [22].

### PCP-related factors

#### *Communication to patients*

Two studies had conflicted findings in regards to the effectiveness of PCP-patient communication and adequate time to follow-up [21, 24]. In one study, patients who had an understanding of their abnormal mammogram results and of the need for follow-up were 3.86 times more likely to attend follow-up (95%CI 1.50–9.96; *P* = 0.006) [24]. In contrast, a small study by Jones et al. found no association between inadequate communication of results and delayed follow-up (aOR = 0.60; 95%CI 0.20–1.83) [21].

Other studies found PCPs had not informed some women of abnormal mammogram results or of the need for follow-up. Casalino et al. found that 5.3% of women had not been informed of their abnormal mammogram within 90 days, [36] and in women that failed to attend 6-monthfollow-up, McCarthy et al. found 13% were not notified of their abnormal mammogram [25]. In women who were non-compliant to complete follow-up, 35% were *not* informed of the need for follow-up compared with 0% of women who were compliant (*P* = 0.008) [17].

Qualitative interviews of 33 low-incomeethnically-diverse women with inadequate follow-up found *all* women were dissatisfied with the lack of information on abnormal mammogram results and recommended follow-up provided by their PCP and some experienced disrespectful behaviour, mistreatment, lack of courtesy, privacy and/or trust in conveying test results and suspicion regarding financial motives of recommending follow-up [40]. Comparatively, women that had adequate follow-up (*n* = 31) cited that communication efforts by PCPs and clinic staff, such as reminder phone calls and letters, were fundamental to their compliance with follow-up [40].

#### *PCPs’ expertise*

Grossman et al. found PCPs who had less clinical experience were less likely to be aware of abnormal mammogram results (aOR = 0.92; 95%CI 0.88–0.97; *P* < 0.05) and less likely to have a follow-up plan (aOR = 0.93; 95%CI 0.87–0.99; *P* < 0.05). However, professional status [Resident or fellow vs attending physician (aOR = 1.10; 95%CI 0.21–5.69)] or number of clinical sessions per week [sessions/week: < 2, 2–2.9, 3–3.9, 4+ (aOR = 0.64; 95%CI 0.12–3.29)], did not impact on follow-up.) [37]. Women that had PCPs with documented evidence of follow-up plans were 2.8 times more likely to receive adequate follow-up (95%CI 1.11–6.98; *P* = 0.029) [24].

### Patient-related factors

Patient-related factors were classified into sociodemographic, psychological or clinical.

#### **Sociodemographic factors**

##### Age

Five studies found older women were more likely to have adequate follow-up (50+ years, [24] 65+ years, [23, 27] and 70+ years), [19, 22] however, two studies had conflicting findings, [19, 35] and two studies found no association between age and adequate follow-up [17, 18, 25].

##### Ethnicity

Jones et al. and Nguyen et al. found African-American and Asian women were more likely to have inadequate follow-up [19, 21]. However, seven studies found no association between ethnicity/race (African-American, Hispanic, Asian, Pacific Islanders, White or other) and follow-up [16–18, 22–25, 37].

##### Income

Three studies found low household income did not influence follow-up (<$USD20K vs ≥ $USD20K, [18, 35] and < $USD10K vs > $USD15K or $USD10K-$USD15K vs > $USD15K), [16] whereas McCarthy et al. found income (≤$USD20K vs > USD$20 K) was associated with non-attendance to 6-monthfollow-up (ARR = 2.1; 95%CI 1.2–3.9). However, this analysis adjusted for all variables, [25] and subsequent analyses only adjusting for relevant variables found this association was no longer significant [18].

##### Health insurance

Seven studies examined the impact of health insurance status (public, [27] private/military, [17, 27] Medicare, [17, 37] Medicaid, [17, 35, 37] managed care, [24] “insurance with full mammogram coverage”, [21] commercial, [37] or not specified) [16] on follow-up. Of these, only managed care was associated with increased likelihood to attend follow-up (aOR = 3.54; 95%CI 1.17–10.66; *P* = 0.026) [24]..

##### Education

Two of six studies found lower educational attainment was associated with inadequate follow-up [19, 27]. Yabroff et al. and Nguyen et al. found women with formal education below high school were less likely to attend follow-up than college graduates or higher (OR = 0.56; 95%CI 0.32–0.98, [27] and Adjusted Hazard Ratio (aHR) = 0.75; 95%CI 0.72–0.78, [19] respectively). High school graduates were also less likely to attend follow-up than college graduates or higher (aHR = 0.86; 95%CI 0.83–0.89) [19]..

##### Marital status

Three individual studies found no association between marital status and follow-up [16, 18, 21].

##### Employment/occupation

Two studies found no association between employment status at time of mammogram and follow-up [18, 21].

#### **Psychological factors**

Qualitative interview of women with inadequate follow-up identified psychological barriers to follow-up, including fatalism, [17] pain/embarrassment, [17, 26] and fear of breast cancer, [40] of losing a breast, [17] and of radiation [26]. While interviews are beneficial in helping provide an understanding of women’s interpretations of their own experiences, quantitative analyses found fear of a breast cancer diagnosis, [17, 18, 24] perceived benefit of mammograms, [18] and mammogram pain/discomfort were not associated with inadequate follow-up [24]. However, the small study by Jones et al. found pain was a significant predictor of inadequate follow-up, with women experiencing painful mammograms 2.8 times (95%CI 1.21–6.47) less likely to attend follow-up compared with women who experienced little/no pain during mammograms [21].

Allen et al. found facilitators of adequate follow up included women’s confidence to advocate for themselves and take responsibility for their self-care [40].

#### **Clinical factors**

##### Health status

Health status did not influence follow-up. While Yabroff et al. found self-reported health was associated with non-attendance to follow-up (“fair/poor/missing” vs “excellent/very good”, OR = 0.60; 95%CI 0.37–0.97), lack of information on missing data prevented conclusive inferences [27]. Burack et al. also found frequency of primary care visits in the previous year was not associated with complete follow-up [35].

##### Family history of breast cancer

Two of five studies found a family history of breast cancer was associated with adequate follow-up [17, 19, 22, 24, 27].

Yabroff et al. found women with a *high* risk family history were 1.65 times (95%CI 1.04–2.62) more likely to attend follow-up than women with low risk, but *medium* risk family history vs no history was not significantly associated with follow-up (OR = 0.84; 95%CI 0.53–1.33) [27]. Likewise, Nguyen et al. found a family history of breast cancer was associated with attendance at follow-up (aHR = 1.05; 95%CI 1.02–1.07) [19]. Three studies found no association between breast cancer family history and follow-up [17, 22, 24].

##### Breast symptoms

Three of four studies found the presence of breast symptoms (e.g. a lump) that occurred incidentally at the time of the screening mammogram influenced follow-up [22, 26, 35, 38]. Schootman et al. found the presence of a lump vs other/no lump was associated with attendance to follow-up (aRR = 2.08; 95%CI 1.18–3.64) [38]. Wernli et al. found women with breast symptoms were less likely to have delayed follow-up within 7 days (aOR = 0.47; 95%CI 0.39–0.56) [22]. Conversely, Burack et al. found no association between breast symptoms and follow-up [35]. Qualitative interview of women with inadequate follow-up by Duijm et al. found 43.8% of women attributed “no breast complaints” for their failure to attend follow-up [26].

A history of mammograms and in particular a history of fewer mammograms (1–2 vs 3–4) was associated with non-attendance to 6-monthfollow-up (aRR = 4.0; 95%CI 1.6–10.4, [18] and aRR = 1.6; 95%CI 1.1–2.3, respectively) [25]. However, Rojas et al. [17] and Poon et al. [24] found no association between prior mammogram history (or prior *abnormal* mammogram history) and follow-up.

## Discussion

Screening mammography is an effective strategy for the early detection of breast cancer and is associated with reduced mortality [2, 45]. However, delays in the follow-up of abnormal mammogram results may compromise the prognostic benefits of screening [4, 5]. This systematic review of 18 articles (reporting on 17 individual studies) examining inadequate follow-up of abnormal mammograms in primary care identified suboptimal follow-up across all included studies, except one study with a paper-based “fail-safe” system for follow-up where patients were tracked and followed up by the radiology department and the clinician only became involved when the patient could not be contacted [37].

Failure to attend recommended 6-month scheduled follow-up for lower risk mammograms (BIRADS®-3) (the most frequently-examined measure of inadequate follow-up) was 27.3, 35.7, 36.8% in three US-based studies, [23, 25, 41] consistent with rates of inadequate follow-up in a non-primary care setting (28%) [46]. However, failure to attend 6-monthfollow-up was significantly higher in the Dutch-based study at 71.6%, [26] which may reflect the reduced role of PCPs in the breast cancer screening regimen in the Netherlands where women are invited for mammogram via nationwide screening programs and PCPs only become involved when the women receives an abnormal result [9]. Failure to attend immediate follow-up (within 3 months) for high risk mammograms (BIRADS®- 4,-5,-0) in US-based studies was also lower (7.2–33%) [21, 25]. Similarly, patients presenting with breast symptoms, [22, 38] extremely dense tissue, [22] or a family history of breast cancer, [19, 27] were more likely to receive adequate follow-up, consistent with studies in non-primary care settings [47–49]. Collectively, these findings suggest providers and/or patients prioritise follow-up in higher risk patients. However, making comparisons between rates of inadequate follow-up across included studies was difficult, particularly over time, due to inconsistencies in definitions of inadequate follow-up, time intervals examined, study design, populations studied and primary care settings across studies.

Individual patient-, PCP- and health system-relatedfactors were found to influence abnormal mammogram follow-up. In particular, women of ethnic minority (African-American, [21] and Asian women) [19] were less likely to have adequate follow-up in primary care. Kaplan et al. also found Latina women in primary care with breast symptoms or an abnormal mammogram (examined collectively, therefore excluded from this review) were less likely to have adequate follow-up, [50] and these discrepancies in the follow-up of ethnic minority women have been found to extend beyond primary care settings to hospital and other non-primary care facilities [51, 52]. Further, given the persistent disparities in later stage breast cancer diagnoses and increased mortality in African-American and Latina women reported across several studies, [53–55] identifying and addressing barriers to the suboptimal follow-up of abnormal mammograms in these populations is imperative in order to improve breast cancer outcomes.

Women with lower education attainment (≤high school graduation) were less likely to receive adequate follow-up in primary care [19, 27]. Moreover, a study examining patient’s understanding of abnormal mammogram results by Karliner et al. found lower education attainment may translate to reduced understanding of the implications of abnormal mammogram results and/or the need for follow-up in some women [56]. Further to this, Yabroff et al. suggested measures to assess abnormal mammogram comprehension and health literacy in women were likely to be beneficial in increasing healthcare utilisation by women [27]. Given lower education attainment has been found to contribute to later stage breast cancer diagnoses, [57, 58] addressing this barrier could improve patient outcomes.

While several studies found women > 50 years were more likely to have adequate follow-up, [19, 22–24, 27] findings were conflicted [19, 35]. Studies not included in this review found inconsistencies between age and abnormal mammogram follow-up: Haas et al. found timely follow-up of abnormal screening or diagnostic mammograms was higher in women > 50 years [59]; whereas Kaplan et al. found age was not a significant predictor of follow-up in women with breast symptoms and/or an abnormal mammogram (examined collectively) [50]. Although the probability that an abnormal mammogram is due to breast cancer increases with age, especially after the age of 50, [60] this observation raises concerns about potential breast cancer diagnosis delays in younger women.

Several important psychological barriers preventing women from attending follow-up, including fear of pain or cancer, [17, 40] pain/embarrassment, [17, 21, 26] and fatalistic beliefs, [17] were identified. Similar barriers have been found in women with inadequate follow-up in non-primary care settings, [49] and in women with abnormal mammogram/breast symptoms (examined collectively) in primary care [61]. Moreover, fear of diagnostic procedures preventing timely follow-up has been found in women with abnormal cervical screening results, [62] and patients with positive fecal occult blood test (FOBT) results [63].

Evidence suggests improved PCP-patient communication may help overcome patient-related barriers to follow-up in primary care and in turn, improve patient outcomes. In particular, several included studies highlighted that effective PCP-patient communication was key to ensuring women understood their abnormal mammogram results and the need for follow-up, [17, 24, 40] consistent with other studies not included in this review [64, 65]. Further, Kerner et al. found African-American women with an abnormal mammogram that had open dialogue with their physician and received clear information about recommended follow-up procedures were more likely to have adequate follow-up across primary and non-primary care [46].

Patient navigator interventions have been found to be effective at addressing patient-related barriers and improving follow-up. Battaglia et al. found patient navigation alone improved timely follow-up in low-income and ethnic minority women with an abnormal mammogram in primary care, [66] however, this study was not included as inadequate follow-up was not specifically addressed. The RCT by Ferrante et al. found women with an abnormal mammogram receiving navigated care in non-primary care not only had improved follow-up, but also reported less anxiety and increased satisfaction with their follow-up care [67]. Further, navigated care was found to be associated with reduced stages of breast and cervical cancer diagnoses [68].

One included RCT by Ell et al. demonstrated the effectiveness of patient navigation with case management and increased PCP-patient education and counselling (the SAFe intervention) in improving follow-up in ethnic minority women in primary care [34]. This intervention and similar models were found to increase abnormal mammogram follow-up in low-income women in non-primary care settings [69, 70]. In particular, the RCT by Maxwell et al. found patient navigation that included emotional support, translation and assistance with overcoming barriers to assessing follow-up effectively improved follow-up in Korean American women attending non-primary care screening centres across California [71]. Further, Llovet et al. indicated the potential of patient navigation and improved PCP-patient communication to address miscommunication of FOBT+ results and fears of diagnostic procedures [63]. Collectively these findings indicate further studies examining these models in abnormal mammogram follow-up in primary care are warranted to test this approach, particularly in ethnic minority women, and examine patient satisfaction and cost-effectiveness [72].

Two included studies illustrated the effectiveness of clinician reminder alerts or alerts to improve abnormal mammogram follow-up in primary care [26, 37]. However, within EHR-based systems, Casalino et al. and Smith et al. found “alert” notifications of a collection of abnormal test results in primary care created information overload due to notifications containing both test results and clinical procedures/policies which in turn contributed to inadequate follow-up [36, 39]. Consistent with these findings, studies across nationwide Veteran Affairs (VA) facilities, [73] and in a non-primary care VA outpatient setting, [74] not included in this review also found PCPs perceived alerts created information overload due the extent of unrelated information sent with test results. Moreover, Hysong et al. and Singh et al. found PCPs perceived that automated EHR alerts would be more efficient if PCPs managed their own alerts so patient’s test results were received in a separate alert from clinical management information and the amount of clinical information received could also be reduced to decrease notification overload [73, 74]. Despite these limitations, Al-Mutairi et al. found only 7.7% of abnormal imagining results, including one abnormal mammogram, were “lost to follow-up” within 4 weeks within a large VA ambulatory clinic with automated EHR-based alerts [75].

Further barriers identified by PCPs included the absence of a reliable method in place to identify patients defaulting follow-up, [25] and PCPs’ difficulty accessing patient records, [26] that need to addressed in order to improve abnormal follow-up in primary care. Further, Casalino et al. and Smith et al. found PCPs perceived uncertainty surrounding their role and responsibility in abnormal mammogram follow-up across different healthcare platforms and with different providers also contributed to inadequate follow-up [36, 39]. Consistent with these findings, another study examining the follow-up of a collection of ambulatory test results found PCPs perceived having “no system in place as a reminder”, “insufficient retrieval systems” or uncertainty of responsibility contributed to delayed follow-up, [76] however, the failure to delineate abnormal mammogram follow-up precluded this study from review. Singh et al. found the absence of standard protocols and procedures in place to manage the follow-up of results was responsible for the inadequate follow-up of abnormal radiology results within an EHR-based primary care system [77]. PCPs perceived improved display, sorting and visualisation of test results within EHR-based alert notifications, that included assigning and displaying who was responsible for follow-up, was needed to improve both the communication and management of test results [73, 74].

Logistical barriers preventing women from accessing follow-up identified, including unavailable medical appointment times, [17, 18, 40] and childcare issues, [17, 40] will need to be addressed to improve abnormal mammogram follow-up. While two RCTs not included in this review indicated that follow-up could be improved if the role of patient navigators included overcoming barriers related to inappropriate appointment scheduling at screening clinics, [71] and in hospital settings, [78] it is likely that changes at an institutional level will also be required to increase access to follow-up. For instance, increasing availability of follow-upcare to evenings and weekends could accommodate women with full-time employment or childcare demands.

This review found the resources offered by managed care insurance translated to improved follow-up in these women, [24] consistent with another study examining abnormal and screening mammogram follow-up collectively [59]. However, we found other forms of private health insurance did not influence follow-up [17, 21, 27, 37]. Reported advantages of managed care including consistent care, [59] and access to more resources or/and less barriers than women without insurance or other forms of insurance, [67, 79] further reinforces the benefits of added support systems in place to improve abnormal mammogram follow-up.

Study strengths include our rigorous systematic review methodology and comprehensive overview of barriers and/or facilitators associated with abnormal mammogram follow-up in primary care. Strict exclusion and inclusion criteria ensured only studies specifically examining inadequate follow-up were included in the review. Moreover, **all** study findings and outcomes were reported from studies relevant to inadequate follow-up in primary care. Subsequently, due to the stringent methodology used, we do not believe this review is subject to selection bias.

Due to heterogeneity in study measures, a narrative review was performed. Additionally, this heterogeneity prevented a feasible evaluation of potential publication bias by quantitative methodology using measures such as forest plots. Subsequently, it can only be postulated as to whether this review was subject to publication bias. That is, there may be bias towards publishing papers that report high rates of inadequate follow-up as these papers highlight the extent of suboptimal abnormal mammogram follow-up in primary care and are clinically relevant to patient care. Conversely, bias to publishing studies with low rates of inadequate follow-up may also occur as these papers help validate breast cancer screening (and abnormal mammogram follow-up) in a primary care setting.

Further, the review was limited by the quality of included studies, with one study at high risk of bias and seven studies at moderate risk. The level of evidence to support factors associated with follow-up also varied. Some studies based findings on self-reported surveys to report PCP and patient perceptions which are subject to recall and/or responder bias and may affect the external reliability of these findings [26, 39, 40]. Other studies used judicious analyses to examine associations and used appropriate methods to select for relevant variables to include in multivariable models, [18, 21, 23, 24, 27, 35, 37] with others adjusting for potentially inappropriate variables in analyses [19, 22, 25]. Moreover, some studies were conducted in large institutions, [17, 21, 34, 37] with others in small clinics, [36] and across both settings [18, 25]. In studies conducted across both public and private facilities, it was also difficult to determine the extent conducted in a primary care setting, however, the decision was made to exclude studies performed < 80% in primary care.

A further study limitation included the generalizability of study findings to countries outside of the US as all studies with the exception of one Dutch-based study published in 1998 were conducted in the US which has unique primary and ambulatory care systems and study populations in terms of ethnicity and socioeconomic status. Further, PCPs in the US are directly involved in promoting breast cancer screening and referring patients for mammograms, as well as organising the follow-up of abnormal mammograms, [80] whereas several developed countries throughout Europe, [81, 82] and in Canada, [12] and Australia, [82] have implemented population-based organized or opportunistic breast cancer screening programs, with invitations for abnormal mammogram follow-up occurring predominantly via automated fail-safe mechanisms. Subsequently, breast cancer screening outside the US is less reliant on primary care for abnormal mammograms follow-up and PCP involvement. Moreover, the relevance of study findings to countries with formalised breast screening programs that operate in parallel to primary care, is also unclear.

## Conclusions

Narrative systematic review predominantly highlighted the suboptimal follow-up of abnormal mammograms in primary care in the US, potentially compromising benefits of breast cancer screening. While EHR-enabled tracking and reminder alerts were demonstrated to be effective in follow-up, patient navigation and case management, with increased PCP-patient communication, may improve follow-up. Additionally, PCP-customisation of alerts, greater accessibility to patient records, and clarifying PCPs’ roles and responsibilities in the follow-up process, and minimising logistical barriers to accessing care such as inconvenient clinic hours are warranted. Overall, addressing factors contributing to inadequate follow-up with targeted interventions, especially in subgroups of women most at risk (ethnic minority and less educated women) could potentially optimize abnormal mammogram follow-up in primary care and improve patient outcomes.

## Supplementary Information

**Additional file 1: Supplementary material 1**. Search strategy

**Additional file 2: Supplementary Table 2**. Summary of Joanna Briggs Institute risk of bias assessments for included studies according to study type

## Data Availability

All data generated or analysed during this study are included in this published article and its supplementary information files.

## Abbreviations

ACR: American College of Radiology
aHR: Adjusted Hazard Ratio
aOR: Adjusted Odds Ratio
BIRADS®: Breast Imaging Reporting and Data System®
EHR: Electronic health record
CI: Confidence interval
JBI: Joanna Briggs Institute
HR: Hazard Ratio
MeSH: Medical Subject Headings
OR: Odds Ratio
PCP: Primary care provider
RCT: Randomised control trial
SAFe: Screening Adherence Follow-up
