# Supplementary material for: Delayed or failure to follow-up abnormal breast cancer screening mammograms in primary care: a systematic review
Source: BMC Cancer. 2021 Apr 7;21:373. doi: 10.1186/s12885-021-08100-3 (PMC8028768; doi:10.1186/s12885-021-08100-3)
Supplement: Supplementary file 1 — Additional file 1: Supplementary material 1. Search strategy [file 12885_2021_8100_MOESM1_ESM.docx]

**Supplementary material 1 – search strategy**

Failure to follow up abnormal test results in primary care search strategies (to October 29, 2020)

Database(s): **Ovid MEDLINE(R) and Epub Ahead of Print, In-Process & Other Non-Indexed Citations and Daily,** from 1946

| **#** | **Searches** | **Results** |
| --- | --- | --- |
| 1 | General practice/ or Family practice/ or Physicians, Family/ or Physicians, Primary Care/ or General practitioners/ or Primary Health Care/ or Ambulatory care/ | 206400 |
| 2 | (General practi* or primary care or primary healthcare or primary health care or family physician* or ambulatory care or outpatient care).ti,ab. | 225091 |
| 3 | 1 or 2 | 320208 |
| 4 | Medical errors/ or Delayed diagnosis/ or Diagnostic Errors/ or Incidental findings/ or Clinical decision-making/ | 80668 |
| 5 | (Error* or delay* or lack or miss or missed or misdiagnos*).ti,ab. | 1384955 |
| 6 | (Follow up or flag up or red flag* or abnormal or trigger*).ti,ab. | 1585116 |
| 7 | (patient safety or quality improvement or diagnostic evaluation).ti,ab. | 71782 |
| 8 | 4 or 5 or 6 or 7 | 2956445 |
| 9 | Diagnostic tests, routine/ or Clinical Laboratory Techniques/ | 34430 |
| 10 | (Test result* or lab* result* or lab* test* or patholog* result* or patholog* test*).ti,ab. | 112712 |
| 11 | Occult Blood/ or (F?ecal occult blood test* or F?ecal immunochemical test* or FOBT).ti,ab. | 7555 |
| 12 | Hematological tests/ or (h?ematologic test* or blood test*).ti,ab. | 29153 |
| 13 | Prostate-specific antigen/ or (Prostate-Specific Antigen or PSA).ti,ab. | 51595 |
| 14 | CA-125 antigen/ or (CA125* or CA-125*).ti,ab. | 10052 |
| 15 | Inflammatory marker*.ti,ab. | 22433 |
| 16 | C-Reactive Protein/ or (C-reactive protein or CRP).ti,ab. | 93203 |
| 17 | Blood Sedimentation/ or (erythrocyte sedimentation rate or ESR).ti,ab. | 34274 |
| 18 | Erythrocyte Indices/ or (Mean corpuscular volume or Mean cell volume or MCV or Mean corpuscular h?emoglobin or Mean cell h?emoglobin or mean cell h?emoglobin concentration or mean corpuscular h?emoglobin concentration or MCHC).ti,ab. | 12724 |
| 19 | Blood Cell Count/ or Erythrocyte Count/ or Leukocyte Count/ or (full blood count or full blood examination or complete blood count or erythrocyte count or red cell count or leucocyte count or white cell count).ti,ab. | 95781 |
| 20 | Anemia/ or Iron/ or Anemia, Iron-Deficiency/ or (an?emia or h?emoglobin or iron).ti,ab. | 472830 |
| 21 | Thrombocytosis/ or (thrombocyt* or platelet count).ti,ab. | 91962 |
| 22 | Hepatitis B/ or Hepatitis C/ or (hepatitis B or hepatitis C).ti,ab. | 162600 |
| 23 | Papillomavirus Infections/ or Human papillomavirus 16/ or Human papillomavirus 18/ or Human papillomavirus 31/ or (human papillomavirus or Human Papilloma Virus or HPV).ti,ab. | 54857 |
| 24 | Papanicolaou Test/ or (pap smear* or pap test* or cervical smear* or papanicolaou test* or cervical screening test*).ti,ab. | 13886 |
| 25 | Mammography/ or Mammogra*.ti,ab. | 40983 |
| 26 | Mass Chest X-Ray/ or Radiography, Thoracic/ or (chest x-ray* or chest xray*).ti,ab. | 53618 |
| 27 | Radiology/ or (radiolog* or imaging).ti,ab. | 1028415 |
| 28 | Ferritins/ or ferritin.ti,ab. | 32896 |
| 29 | Transferrin/ or transferrin.ti,ab. | 34414 |
| 30 | Calcium/ or (calcium or hypercalc?emia).ti,ab. | 508007 |
| 31 | (skin biops* or skin excision* or derma* biops* or derma* excision* or excision* biops*).ti,ab. | 22841 |
| 32 | Tomography, X-Ray Computed/ or (CT scan or CAT scan or computeri?ed tomograph* or computed axial tomograph*).ti,ab. | 425677 |
| 33 | ultrasound.ti,ab. | 249374 |
| 34 | (Cancer* or Carcinoma* or Neoplasm* or cancer screen*).ti,ab. or neoplasms/ | 2448947 |
| 35 | 9 or 10 or 11 or 12 or 13 or 14 or 15 or 16 or 17 or 18 or 19 or 20 or 21 or 22 or 23 or 24 or 25 or 26 or 27 or 28 or 29 or 30 or 31 or 32 or 33 or 34 | 5213539 |
| 36 | 3 and 8 and 35 | 10292 |
| 37 | limit 36 to english language | 9425 |
| 38 | limit 37 to yr="1990 -Current" | 9130 |

Database(s): **Embase Classic+Embase,** from 1947

| **#** | **Searches** | **Results** |
| --- | --- | --- |
| 1 | General practice/ or Family practice/ or Physicians, Family/ or Physicians, Primary Care/ or General practitioners/ or Primary Health Care/ or Ambulatory care/ | 273984 |
| 2 | (General practi* or primary care or primary healthcare or primary health care or family physician* or ambulatory care or outpatient care).ti,ab. | 302354 |
| 3 | 1 or 2 | 424101 |
| 4 | Medical errors/ or Delayed diagnosis/ or Diagnostic Errors/ or Incidental findings/ or Clinical decision-making/ | 149328 |
| 5 | (Error* or delay* or lack or miss or missed or misdiagnos*).ti,ab. | 1934184 |
| 6 | (Follow up or flag up or red flag* or abnormal or trigger*).ti,ab. | 2431668 |
| 7 | (patient safety or quality improvement or diagnostic evaluation).ti,ab. | 108824 |
| 8 | 4 or 5 or 6 or 7 | 4333154 |
| 9 | Diagnostic tests, routine/ or Clinical Laboratory Techniques/ | 83266 |
| 10 | (Test result* or lab* result* or lab* test* or patholog* result* or patholog* test*).ti,ab. | 265138 |
| 11 | Occult Blood/ or (F?ecal occult blood test* or F?ecal immunochemical test* or FOBT).ti,ab. | 13855 |
| 12 | Hematological tests/ or (h?ematologic test* or blood test*).ti,ab. | 38231 |
| 13 | Prostate-specific antigen/ or (Prostate-Specific Antigen or PSA).ti,ab. | 99818 |
| 14 | CA-125 antigen/ or (CA125* or CA-125*).ti,ab. | 21947 |
| 15 | Inflammatory marker*.ti,ab. | 39227 |
| 16 | C-Reactive Protein/ or (C-reactive protein or CRP).ti,ab. | 225461 |
| 17 | Blood Sedimentation/ or (erythrocyte sedimentation rate or ESR).ti,ab. | 81994 |
| 18 | Erythrocyte Indices/ or (Mean corpuscular volume or Mean cell volume or MCV or Mean corpuscular h?emoglobin or Mean cell h?emoglobin or mean cell h?emoglobin concentration or mean corpuscular h?emoglobin concentration or MCHC).ti,ab. | 22356 |
| 19 | Blood Cell Count/ or Erythrocyte Count/ or Leukocyte Count/ or (full blood count or full blood examination or complete blood count or erythrocyte count or red cell count or leucocyte count or white cell count).ti,ab. | 189673 |
| 20 | Anemia/ or Iron/ or Anemia, Iron-Deficiency/ or (an?emia or h?emoglobin or iron).ti,ab. | 770697 |
| 21 | Thrombocytosis/ or (thrombocyt* or platelet count).ti,ab. | 159311 |
| 22 | Hepatitis B/ or Hepatitis C/ or (hepatitis B or hepatitis C).ti,ab. | 255090 |
| 23 | Papillomavirus Infections/ or Human papillomavirus 16/ or Human papillomavirus 18/ or Human papillomavirus 31/ or (human papillomavirus or Human Papilloma Virus or HPV).ti,ab. | 70997 |
| 24 | Papanicolaou Test/ or (pap smear* or pap test* or cervical smear* or papanicolaou test* or cervical screening test*).ti,ab. | 25362 |
| 25 | Mammography/ or Mammogra*.ti,ab. | 61654 |
| 26 | Mass Chest X-Ray/ or Radiography, Thoracic/ or (chest x-ray* or chest xray*).ti,ab. | 193200 |
| 27 | Radiology/ or (radiolog* or imaging).ti,ab. | 1504092 |
| 28 | Ferritins/ or ferritin.ti,ab. | 57480 |
| 29 | Transferrin/ or transferrin.ti,ab. | 48859 |
| 30 | Calcium/ or (calcium or hypercalc?emia).ti,ab. | 626376 |
| 31 | (skin biops* or skin excision* or derma* biops* or derma* excision* or excision* biops*).ti,ab. | 37992 |
| 32 | Tomography, X-Ray Computed/ or (CT scan or CAT scan or computeri?ed tomograph* or computed axial tomograph*).ti,ab. | 173719 |
| 33 | (Cancer* or Carcinoma* or Neoplasm* or cancer screen*).ti,ab. or neoplasms/ | 3316885 |
| 34 | ultrasound.ti,ab. | 391893 |
| 35 | 9 or 10 or 11 or 12 or 13 or 14 or 15 or 16 or 17 or 18 or 19 or 20 or 21 or 22 or 23 or 24 or 25 or 26 or 27 or 28 or 29 or 30 or 31 or 32 or 33 or 34 | 7202800 |
| 36 | 3 and 8 and 35 | 19387 |
| 37 | limit 36 to english language | 18313 |
| 38 | limit 37 to yr="1990 -Current" | 17993 |
| 39 | conference*.pt. | 4672180 |
| 40 | 38 not 39 | 10309 |

Database(s): **EBM Reviews - Cochrane Central Register of Controlled Trials**September 2020**, EBM Reviews - Cochrane Database of Systematic Reviews**, from 2005

| **#** | **Searches** | **Results** |
| --- | --- | --- |
| 1 | General practice/ or Family practice/ or Physicians, Family/ or Physicians, Primary Care/ or General practitioners/ or Primary Health Care/ or Ambulatory care/ | 9850 |
| 2 | (General practi* or primary care or primary healthcare or primary health care or family physician* or ambulatory care or outpatient care).ti,ab. | 30292 |
| 3 | 1 or 2 | 33744 |
| 4 | Medical errors/ or Delayed diagnosis/ or Diagnostic Errors/ or Incidental findings/ or Clinical decision-making/ | 701 |
| 5 | (Error* or delay* or lack or miss or missed or misdiagnos*).ti,ab. | 100148 |
| 6 | (Follow up or flag up or red flag* or abnormal or trigger*).ti,ab. | 235788 |
| 7 | (patient safety or quality improvement or diagnostic evaluation).ti,ab. | 4906 |
| 8 | 4 or 5 or 6 or 7 | 320313 |
| 9 | Diagnostic tests, routine/ or Clinical Laboratory Techniques/ | 355 |
| 10 | (Test result* or lab* result* or lab* test* or patholog* result* or patholog* test*).ti,ab. | 22865 |
| 11 | Occult Blood/ or (F?ecal occult blood test* or F?ecal immunochemical test* or FOBT).ti,ab. | 1192 |
| 12 | Hematological tests/ or (h?ematologic test* or blood test*).ti,ab. | 5890 |
| 13 | Prostate-specific antigen/ or (Prostate-Specific Antigen or PSA).ti,ab. | 8087 |
| 14 | CA-125 antigen/ or (CA125* or CA-125*).ti,ab. | 952 |
| 15 | Inflammatory marker*.ti,ab. | 5731 |
| 16 | C-Reactive Protein/ or (C-reactive protein or CRP).ti,ab. | 23105 |
| 17 | Blood Sedimentation/ or (erythrocyte sedimentation rate or ESR).ti,ab. | 3955 |
| 18 | Erythrocyte Indices/ or (Mean corpuscular volume or Mean cell volume or MCV or Mean corpuscular h?emoglobin or Mean cell h?emoglobin or mean cell h?emoglobin concentration or mean corpuscular h?emoglobin concentration or MCHC).ti,ab. | 816 |
| 19 | Blood Cell Count/ or Erythrocyte Count/ or Leukocyte Count/ or (full blood count or full blood examination or complete blood count or erythrocyte count or red cell count or leucocyte count or white cell count).ti,ab. | 5506 |
| 20 | Anemia/ or Iron/ or Anemia, Iron-Deficiency/ or (an?emia or h?emoglobin or iron).ti,ab. | 42963 |
| 21 | Thrombocytosis/ or (thrombocyt* or platelet count).ti,ab. | 11375 |
| 22 | Hepatitis B/ or Hepatitis C/ or (hepatitis B or hepatitis C).ti,ab. | 16206 |
| 23 | Papillomavirus Infections/ or Human papillomavirus 16/ or Human papillomavirus 18/ or Human papillomavirus 31/ or (human papillomavirus or Human Papilloma Virus or HPV).ti,ab. | 3425 |
| 24 | Papanicolaou Test/ or (pap smear* or pap test* or cervical smear* or papanicolaou test* or cervical screening test*).ti,ab. | 1144 |
| 25 | Mammography/ or Mammogra*.ti,ab. | 2554 |
| 26 | Mass Chest X-Ray/ or Radiography, Thoracic/ or (chest x-ray* or chest xray*).ti,ab. | 2457 |
| 27 | Radiology/ or (radiolog* or imaging).ti,ab. | 51843 |
| 28 | Ferritins/ or ferritin.ti,ab. | 3983 |
| 29 | Transferrin/ or transferrin.ti,ab. | 2270 |
| 30 | Calcium/ or (calcium or hypercalc?emia).ti,ab. | 24453 |
| 31 | (skin biops* or skin excision* or derma* biops* or derma* excision* or excision* biops*).ti,ab. | 1165 |
| 32 | Tomography, X-Ray Computed/ or (CT scan or CAT scan or computeri?ed tomograph* or computed axial tomograph*).ti,ab. | 8963 |
| 33 | ultrasound,ti.ab. | 0 |
| 34 | (Cancer* or Carcinoma* or Neoplasm* or cancer screen*).ti,ab. or neoplasms/ | 171896 |
| 35 | 9 or 10 or 11 or 12 or 13 or 14 or 15 or 16 or 17 or 18 or 19 or 20 or 21 or 22 or 23 or 24 or 25 or 26 or 27 or 28 or 29 or 30 or 31 or 32 or 33 or 34 | 347454 |
| 36 | 3 and 8 and 35 | 2001 |
| 37 | limit 36 to english language [Limit not valid in CDSR; records were retained] | 1307 |
| 38 | limit 37 to yr="1990 -Current" | 1288 |

**CINAHL Complete (EBSCOhost)**

| **#** | **Query** | **Results** |
| --- | --- | --- |
| S1 | (MH “Family practice”) OR (MH “Primary health care”) OR (MH “Physicians, family”) OR (MH “Ambulatory care”) | 110,719 |
| S2 | TI ( General practi* OR primary care OR primary healthcare OR primary health care OR family physician* OR ambulatory care OR outpatient care ) | 56,090 |
| S3 | AB ( General practi* OR primary care OR primary healthcare OR primary health care OR family physician* OR ambulatory care OR outpatient care ) | 108,618 |
| S4 | (MH “Diagnostic errors”) OR (MH “Diagnosis, delayed”) OR (MH “Decision making, clinical”) OR (MH “Patient safety”) OR (MH “Quality improvement”) | 158,289 |
| S5 | TI ( Error* OR delay* OR lack OR miss OR missed OR misdiagnos* OR Follow up OR flag up OR red flag* OR abnormal OR trigger OR patient safety or quality improvement or diagnostic evaluation) | 107,075 |
| S6 | AB ( Error* OR delay* OR lack OR miss OR missed OR misdiagnos* OR Follow up OR flag up OR red flag* OR abnormal OR trigger OR patient safety or quality improvement or diagnostic evaluation) | 584,530 |
| S7 | (MH “Diagnostic tests, routine”) OR (MH “Occult blood”) OR (MH “Hematological tests”) OR (MH “Prostate-Specific Antigen”) OR (MH “C-Reactive Protein”) OR (MH “Blood Sedimentation”) OR (MH “Erythrocyte Indices”) OR (MH “Blood Cell Count”) OR (MH “Leucocyte count”) OR (MH “Anemia”) OR (MH “Anemia, Iron Deficiency”) OR (MH “Iron”) OR (MH “Thrombocytosis”) OR (MH “Hepatitis B”) OR (MH “Hepatitis C”) OR (MH “Papillomaviruses”) OR (MH “Cervical Smears”) OR (MH “Mammography”) OR (MH “Radiography, Thoracic”) OR (MH “Ferritin”) OR (MH “Transferrin”) OR (MH “Calcium”) OR (MH “Ultrasonography”) OR (MH “Tomography, x-ray computed”) OR (MH “Neoplasms”) OR (MH “Cancer screening”) | 335,574 |
| S8 | TI (Test result* OR lab* result* OR lab* test* OR patholog* result* OR patholog* test* OR F?ecal occult blood test* OR F?ecal immunochemical test* OR FOBT OR h?ematologic test* OR blood test* OR Prostate-Specific Antigen OR PSA OR CA125* OR CA-125* OR Inflammatory marker* OR C-reactive protein OR CRP OR erythrocyte sedimentation rate OR ESR OR Mean corpuscular volume OR Mean cell volume OR MCV OR Mean corpuscular h?emoglobin OR Mean cell h?emoglobin OR mean cell h?emoglobin concentration OR mean corpuscular h?emoglobin concentration OR MCHC OR full blood count OR full blood examination OR complete blood count OR erythrocyte count OR red cell count OR leucocyte count OR white cell count OR an?emia OR h?emoglobin OR iron OR thrombocyt* OR platelet count OR hepatitis B OR hepatitis C OR human papillomavirus OR Human Papilloma Virus OR HPV OR pap smear* OR pap test* OR cervical smear* OR papanicolaou test* OR cervical screening test* OR Mammogra* OR chest x-ray* OR chest xray* OR radiolog* OR imaging OR ferritin OR transferrin OR calcium OR hypercalc?emia OR skin biops* OR skin excision* OR derma* biops* OR derma* excision* OR excision* biops* OR CT scan OR CAT scan OR computeri?ed tomograph* OR computed axial tomograph* OR ultrasound OR Cancer* OR Carcinoma* OR Neoplasm* OR cancer screening) | 495,826 |
| S9 | AB (Test result* OR lab* result* OR lab* test* OR patholog* result* OR patholog* test* OR F?ecal occult blood test* OR F?ecal immunochemical test* OR FOBT OR h?ematologic test* OR blood test* OR Prostate-Specific Antigen OR PSA OR CA125* OR CA-125* OR Inflammatory marker* OR C-reactive protein OR CRP OR erythrocyte sedimentation rate OR ESR OR Mean corpuscular volume OR Mean cell volume OR MCV OR Mean corpuscular h?emoglobin OR Mean cell h?emoglobin OR mean cell h?emoglobin concentration OR mean corpuscular h?emoglobin concentration OR MCHC OR full blood count OR full blood examination OR complete blood count OR erythrocyte count OR red cell count OR leucocyte count OR white cell count OR an?emia OR h?emoglobin OR iron OR thrombocyt* OR platelet count OR hepatitis B OR hepatitis C OR human papillomavirus OR Human Papilloma Virus OR HPV OR pap smear* OR pap test* OR cervical smear* OR papanicolaou test* OR cervical screening test* OR Mammogra* OR chest x-ray* OR chest xray* OR radiolog* OR imaging OR ferritin OR transferrin OR calcium OR hypercalc?emia OR skin biops* OR skin excision* OR derma* biops* OR derma* excision* OR excision* biops* OR CT scan OR CAT scan OR computeri?ed tomograph* OR computed axial tomograph* OR ultrasound OR Cancer* OR Carcinoma* OR Neoplasm* OR cancer screening) | 654,715 |
| S10 | S1 OR S2 OR S3 | 183,734 |
| S11 | S4 OR S5 OR S6 | 760,522 |
| S12 | S7 OR S8 OR S9 | 1,009,648 |
| S13 | S10 AND S11 AND S12 | 5,709 |
| S14 | S10 AND S11 AND S12  Limiters - Published Date: 19900101-20201131; English Language | 5,606 |
