# Supplementary material for: Delayed or failure to follow-up abnormal breast cancer screening mammograms in primary care: a systematic review
Source: BMC Cancer. 2021 Apr 7;21:373. doi: 10.1186/s12885-021-08100-3 (PMC8028768; doi:10.1186/s12885-021-08100-3)
Supplement: Supplementary file 2 — Additional file 2: Supplementary Table 2. Summary of Joanna Briggs Institute risk of bias assessments for included studies according to study type [file 12885_2021_8100_MOESM2_ESM.pdf]

Supplementary Table 2: Summary of Joanna Briggs Institute risk of bias assessments for included studies according to study type

Study (first author)	Year of publication	Questionnaire responses according to respective study type												Score (%)	Risk of bias
		**Q1**	**Q2**	**Q3**	**Q4**	**Q5**	**Q6**	**Q7**	**Q8**	**Q9**	**Q10**	**Q11**	**Q12**		
Analytical cross-sectional															
Rojas et al.	1996	Yes	Yes	Yes	No	No	No	Yes	No	-	-	-	-	50	high
Webber et al.	1996	No	No	Yes	Yes	Yes	Yes	Yes	Yes	-	-	-	-	75	moderate
McCarthy et al.	1996	Yes	Yes	Yes	Yes	Yes	Yes	Yes	Yes	-	-	-	-	100	low
Yabroff et al.	2004	Yes	Yes	Yes	Yes	Yes	Yes	Yes	Yes	-				100	low
Case-control															
Grossman et al.	2010	Yes	Yes	Yes	Yes	Yes	Yes	Yes	Yes	Yes	Yes	-	-	100	low
Cohort															
McCarthy et al.	1996	Yes	Yes	Yes	Yes	Yes	Yes	Yes	Yes	Yes	Yes	Yes	-	100	low
Duijm et al.	1998	NA[a]	NA	Yes	No	NA	Yes	Yes	Yes	No	NA	NA	-	67	moderate
Burack et al.	2000	Unclear	Yes	Yes	Yes	Yes	Unclear	Yes	NA	NA	NA	Yes	-	75	moderate
Schootman et al.	2000	Yes	Yes	Yes	Yes	Yes	Yes	Yes	Yes	No	NA	Yes	-	91	low
Poon et al.	2004	Unclear	Yes	Yes	Yes	Yes	Yes	Yes	Yes	No	Unclear	Yes	-	73	moderate
Jones et al.	2005	Yes	Yes	Yes	Yes	Yes	Yes	Yes	Yes	No	Unclear	Yes	-	81.8	low
Schootman et al.	2007	Yes	Unclear	Yes	No	NA	Yes	Yes	Yes	NA	NA	Yes	-	75	moderate
Casalino et al.	2009	Unclear	Yes	Yes	No	No	NA	Yes	Yes	NA	NA	Yes	-	66.7	moderate
Wernli et al.	2011	Yes	Yes	Yes	Yes	Yes	Yes	Yes	Yes	Yes	Yes	Yes	-	100	low
Nguyen et al.	2017	Yes	Yes	Yes	Yes	Yes	Yes	Yes	Yes	Yes	Yes	Yes	-	100	low
Qualitative															
Allen et al.	2008	Yes	Yes	Yes	Yes	Yes	Yes	No	Yes	Yes	Yes	-	-	90	low
Smith et al.	2018	Yes	Yes	Yes	Yes	Yes	Unclear	Yes	Unclear	Yes	Yes	-	-	80	moderate
Randomised control trial															
Ell et al.	2007	Yes	Yes	Yes	Unclear	NA	Yes	Yes	Yes	Yes	Yes	Yes	Yes	91	low

[a]Not applicable
